# The timing of urinary catheter removal after gynecologic surgery: A meta-analysis of randomized controlled trials

**DOI:** 10.1097/MD.0000000000018710

**Published:** 2020-01-10

**Authors:** Hui Huang, Li Dong, Lan Gu

**Affiliations:** a Department of Nursing, The Second Affiliated Hospital of Soochow University, Suzhou; b Department of Hepatobiliary and Pancreatic Surgery, the First Affiliated Hospital, Zhejiang University School of Medicine, Hangzhou; c Department of Senior Cadres Ward, The First Affiliated Hospital of Soochow University, Suzhou, China.

**Keywords:** gynecologic surgery, urinary catheter removal time, urinary infection, urinary retention

## Abstract

The present study aimed to assess the effect of removing an indwelling urinary catheter at different times on urinary retention and urinary infection in patients undergoing gynecologic surgery.

Electronic databases including PubMed, EMbase, the Cochrane Central Register of Controlled Trials, and Ovid from inception to June 2018 were searched. Relevant randomized controlled trials (RCTs) of removal the indwelling urinary catheter in different time were included.

Eight RCTs were included. Data were analyzed by RevMan 5.3 version. There was significant difference in urinary retention (relative risk [RR] 2.46, 95% confidence intervals [CIs] 1.10–5.53), *P* = .03) between the ≤6 hours and >6 hours indwelling urinary catheter removal groups, while no significant differences were found in the gynecologic surgery excluded the vaginal surgery group and vaginal surgery group. When compared with >6 hours indwelling urinary catheter removal group, the incidence of urinary infection was significantly reduced at the ≤6 hours removal group (RR = 0.66, 95% CI 0.48–0.89, *P* = .007). The urinary catheter removal time at ≤6 hours also significantly reduced the incidence of urinary retention (RR = 5.06, 95%CI 1.74–14.69, *P* = .003), and did not statistically increase the incidence of urinary infection (RR = 0.30, 95%CI 0.08 to 1.20, *P* = .09), compared with immediate urinary catheter removal after surgery.

Removal time of the urinary catheter at ≤6 hours postoperatively seems to be more beneficial than immediate or >6 hours for patients undergoing gynecologic surgery which excluded the vaginal surgery.

## 1 Introduction

Gynecologic surgeries including hysterectomy and cesarean sections are usually performed for the patients with pregnancy and various benign diseases.^[1]^ The placement of an indwelling urinary catheter before gynecologic surgeries is a standard method for preventing bladder injury during operation and postoperative urinary retention.^[2]^ Previous studies more focused on the surgical management during gynecologic surgery.^[3,4]^ An indwelling urinary catheter, which involves an invasive procedure, often results in many patients suffering urinary tract infections (UTI). 70% to 80% of catheter-associated UTI are caused by indwelling catheters.^[5]^ The timing of indwelling urinary catheter removal is the most important risk factor for UTI.^[6]^ It has been established that urinary catheterization increases the risk of infection by 5% to 10% per day of use.^[7]^ At the same time, indwelling urinary catheterization is associated with an increased UTI in patients with gynecologic surgery.^[8]^ Dunn et al^[9]^ reported that immediate removal of urinary catheter after gynecologic surgery significantly reduced UTI-associated pain, whereas Chai et al^[10]^ found that early removal of urinary catheter following total abdominal hysterectomy, compared with the delayed removal group, increased the number of urinary retention episodes which required re-catheterization. A meta-analysis conducted by Zhang et al^[11]^ suggested that catheter removal immediately combined with postoperative urination monitoring might be a good method for patients undergoing gynecologic surgery when compared with the delayed catheter removal group, which focused a delay of 24 hours or more than or close to 24 hours. But Ahmed et al^[12]^ proposed that the more reasonable time of the urinary catheter removal was at 6 hours after operation than immediate or 24 hours after operation in patients with total abdominal hysterectomy. Therefore, the duration of an indwelling urine catheter in patients undergoing gynecologic surgery remains controversial. This study aims to identify the appropriate timing of removing the indwelling urinary catheter in patients undergoing gynecologic surgery through a meta-analysis.

## 2 Methods

### 2.1 Study design

The patient consent and ethical approval for the study were not applicable since the current study is a meta-analysis and all data was collected from published articles.

Meta-analysis was conducted to summarize the results of the included studies, and this study complied with the guidelines in the systematic reviews and meta-analysis of randomized controlled trials (RCT) in preferred reporting items for systematic reviews and meta-analyses statement.^[13]^

### 2.2 Inclusion criteria and exclusion criteria

Studies were included if they met the following criteria:

(1) RCT;
(2) The times of catheter removal involving immediate, within 6 hours (≤6 hours), >6 hours after surgery, and without clamping the urinary tube before extubation;
(3) Outcomes including the incidence of urinary retention and UTI after extubation. The definition of urinary retention was lack of spontaneous micturition 6 hours after catheter removal or post-voiding residual volume exceeding 200 mL measured by transabdominal ultrasound.^[14]^ The diagnosis of UTI was based on the significant bacteria (determined by quantitative urine culture yielding at least 10 colony-forming units of an identified single uropathogen per milliliter)^[7]^ and/or at least 1 of the following symptoms: fever, dysuria, increased frequency of urination, urinary urgency, suprapubic pain, and burning micturition.^[14]^ All patients had postoperative urine culture done in order to confirm the UTI;
(4) Study participants were patients undergoing gynecologic surgery including endoscopic surgery and those aged over 18 years. Exclusion criteria included animal studies, participants with malignant tumors or complicated gynecologic surgery.

### 2.3 Search strategy

We performed a comprehensive search of PubMed, Embase, the Cochrane Central Register of Controlled Trials, and Ovid database from inception to June 30, 2018 to obtain studies meeting the eligibility criteria. Terms used included “urinary catheter” or “indwelling urinary catheter” and “remove” or “removal” or “extubation” or “extraction” or “duration of urinary catheterization” or “time of urinary catheterization” and “surgery” or “operation” or “postoperative.” Systematic reviews and citation search of relevant published studies were also used to locate relevant studies that may have been missed in the strategy described above.

### 2.4 Data extraction and quality assessment

Each of the 2 authors screened studies through the titles and abstracts independently to confirm whether the study met the inclusion criteria. After confirming the eligibility of studies, 2 authors independently extracted relevant data, including author, year of publication, country, type of gynecologic surgery, intervention information, and outcomes. An arbitrator was consulted if there was disagreement between the 2 authors.

The quality of included studies was assessed using the Cochrane collaboration risk of bias tool.^[15]^ There are 6 domains in this tool:

(1) Random sequence generation;
(2) Allocation concealed;
(3) Blinding of participants/personnel;
(4) Blinding of outcome assessment;
(5) Incomplete outcome data;
(6) Selective reporting, and other bias.

Every domain for included studies was evaluated as low, unclear, or high risk of bias. Discrepancies about the quality assessment of included studies were resolved by consensus.

### 2.5 Statistical analysis

Statistical analyses were performed using Review Manager 5.3 software. The relative risk, with corresponding 95% confidence intervals (CIs), was considered as the effect estimate for all included studies. Heterogeneity was evaluated by testing the clinical characteristics of the enrolled studies as well as by formal statistical testing using chi-square and *I*^*2*^ tests. If *P* > .1 and *I*^*2*^ < 50%, a fixed-effect model was used; otherwise, a random-effect model was used. For all statistical analyses, a value of *P* < .05 was considered to indicate statistical significance, and all tests were 2-sided.

## 3 Results

### 3.1 Selection and study characteristics

**Figure 1:**
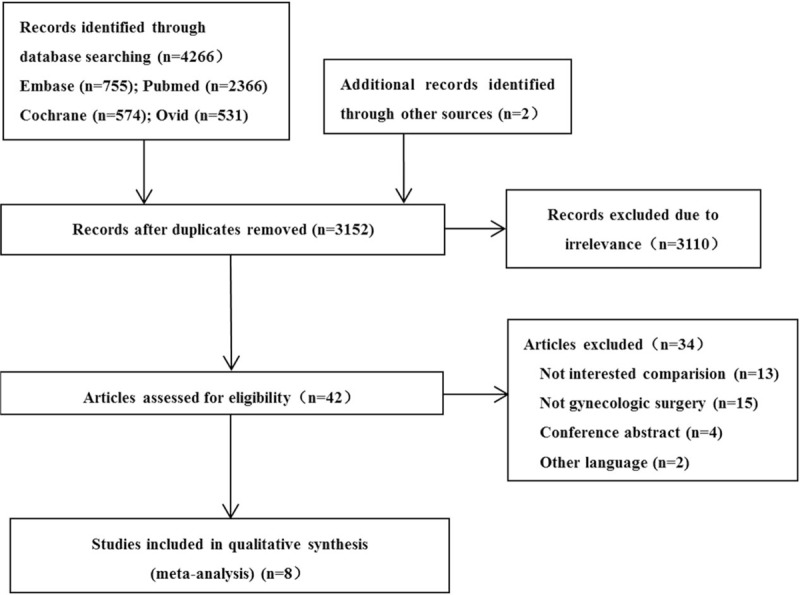
Flow chart of search results.

A total of 4266 studies were obtained by searching through the databases and 2 additional records were identified through other sources. After removing duplicates and reviewing the titles and abstracts, 42 articles were potentially relevant and the full texts of remaining studies were examined in detail; only 8 papers^[1,12,14,16–20]^ satisfied all the inclusion criteria and were therefore included in this review. Flow chart of search results is shown in Figure 1 and characteristics of included studies are presented in Table 1.

**Table 1:**
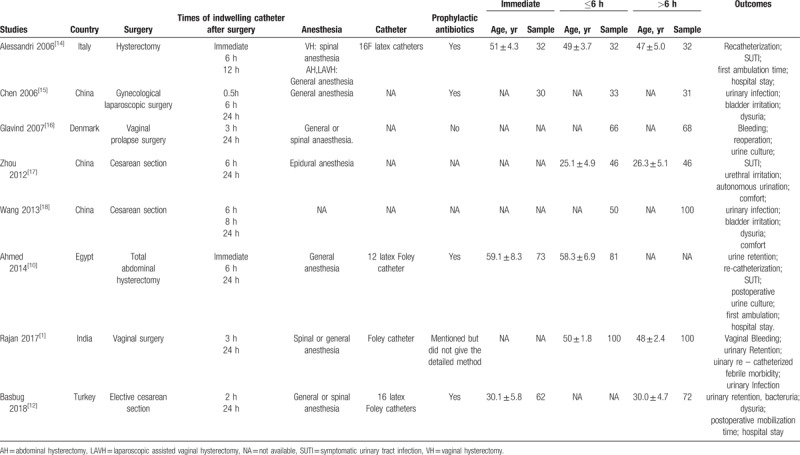
Characteristics of the included studies.

**Figure 2:**
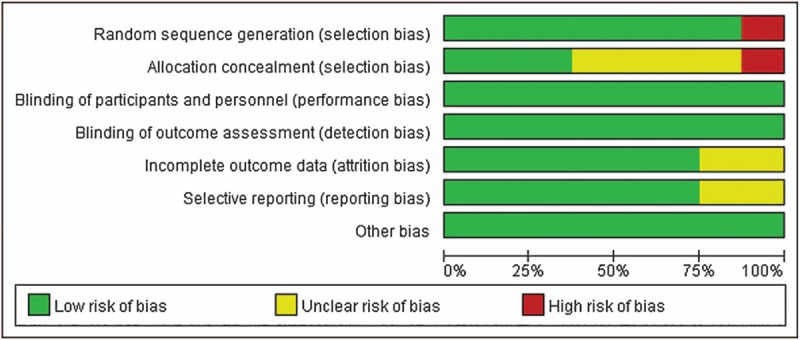
Risk of bias graph of included studies.

**Figure 3:**
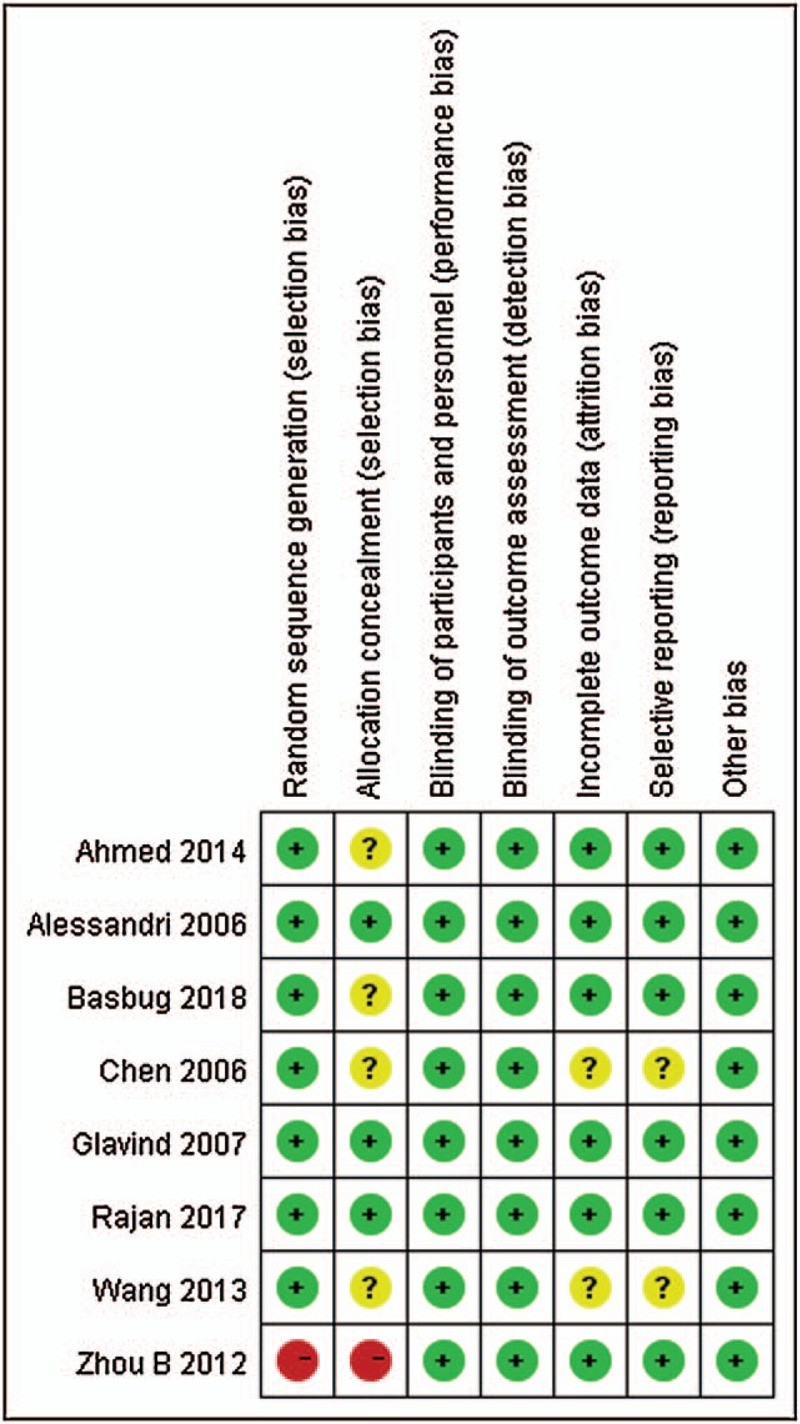
Risk of bias summary of included studies.

The graphs of biases existing in the included studies are shown in Figures 2 and 3. Of these, the study by Alessandri et al,^[16]^ Glavind et al,^[18]^ and Rajan et al^[1]^ was judged to be of low risk in all domains. The study by Ahmed et al^[12]^ and Basbug et al^[14]^ also showed low risk except unclear risk in allocation concealed domain. The study by Zhou et al^[19]^ showed high risk of random sequence generation and allocation concealed, the remaining domains were all low risk. The study conducted by Chen^[17]^ and Wang et al^[20]^ showed unclear risk of allocation concealed, incomplete outcome data and selective reporting.

### 3.2 Comparison of incidences of urinary retention and UTI between ≤6 hours and >6 hours catheter removal after surgery

**Figure 4:**
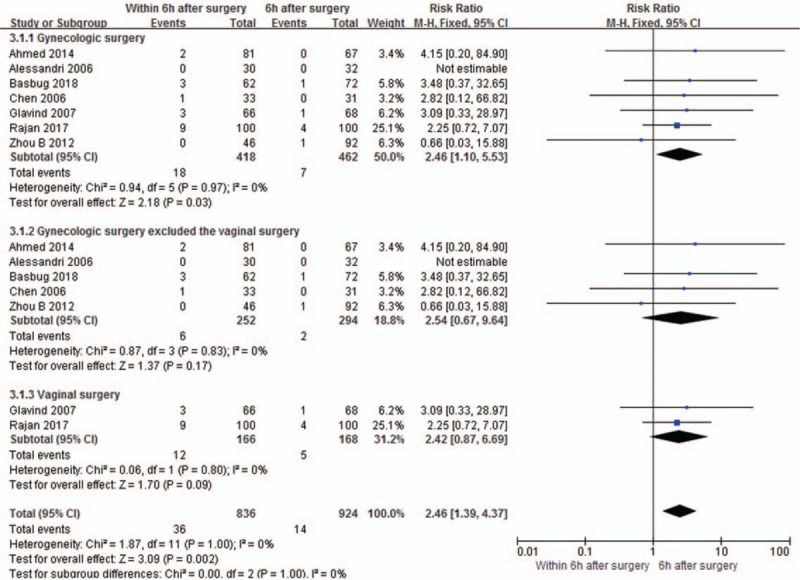
Comparison of the incidences of the urinary retention between catheter extubation time ≤6 h group and >6 h group after surgery.

The incidences of urinary retention were reported in all the included studies.^[1,12,14,16–20]^ No heterogeneity was demonstrated in the pooled result (*I*^*2*^ = 0%). The result of analysis using a fixed-effect model showed significant difference of incidences of urinary retention between ≤6 hours and >6 hours catheter removal groups (relative risk [RR] 2.46, 95% CI 1.10–5.53, *P* = .03, Fig. 4). However, in the subgroup analysis, no significant differences were found in the gynecologic surgery excluded the vaginal surgery group and vaginal surgery group.

**Figure 5:**
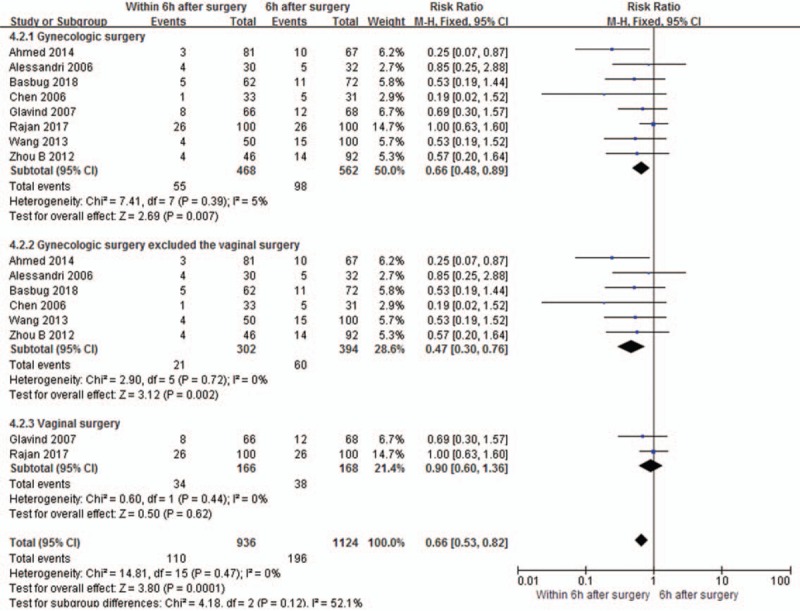
Comparison of the incidences of the urinary tract infection between catheter extubation time ≤6 h group and >6 h group after surgery.

The incidences of UTI were reported in all the included studies.^[1,12,14,16–20]^ A forest plot showed that ≤6 hours extubation after surgery reduced the risk of UTI compared with >6 hours catheter removal after surgery (RR 0.66, 95%CI 0.48–0.89, *P* = .007, Fig. 5), without significant heterogeneity (*I*^*2*^ = 5%). In the subgroup analysis, the results showed that there is significant difference in the gynecologic surgery excluded the vaginal surgery group (RR 0.47, 95%CI 0.30–0.76, *P* = .002, Fig. 5). According to the fail-safe number (Nfs_0.05_) = (Σ*Z*/1.64)^2^-*S* (*Z* representing the *Z* value of each single study; *S* representing the number of all enrolled studies) to calculate Nfs_0.05_, the Nfs_0.05_ was 13, indicating that another 13 negative studies would be needed to reverse this result. Thus, the result of this review as it relates to incidence of UTI is stable. No significant difference in vaginal surgery group (RR 0.90, 95%CI 0.60–1.36, *P* = .62, Fig. 5).

### 3.3 Comparison of incidences of urinary retention and UTI between ≤6 hours extubation group and immediate extubation group after surgery

**Figure 6:**
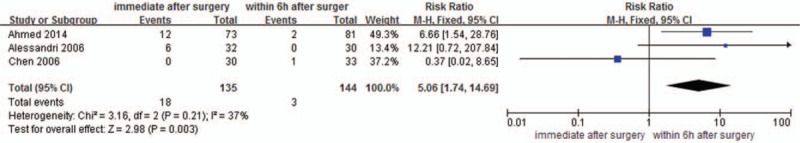
Comparison of the incidences of the urinary retention between catheter extubation time ≤6 h group and immediate extubation after surgery.

Three studies^[12,16,17]^ reported the incidences of urinary retention between immediate and ≤6 hours extubation after surgery. No further data could be obtained. Therefore, 3 studies were included in the meta-analysis. The overall summary RR using the fixed-effects model was 5.06, 95% CI 1.74–14.69, *P* = .003 (Fig. 6), suggesting immediate extubation after surgery increased the incidence of urinary retention. The Nfs_0.05_ regarding urinary retention was 10, which indicated that the result was stable.

**Figure 7:**
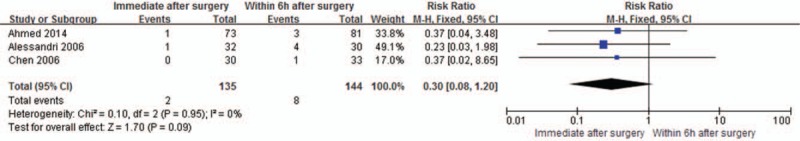
Comparison of the incidences of urinary tract infection between catheter extubation time ≤6 h group and immediate extubation after surgery.

Three studies^[12,16,17]^ reported the incidences of UTI with respect to immediate extubation and ≤6 hours extubation after surgery. There was no significant difference between the incidences of UTI of immediate and ≤6 hours extubation groups after surgery (RR 0.30, 95%CI 0.08–1.20, *P* = .09, Fig. 7), and there was significant heterogeneity (*I*^*2*^ = 0%).

## 4 Discussion

Based on RCT on urinary catheter removal after gynecologic surgery published in English which were screened, the articles were divided into 3 groups, immediate, within 6 hours and longer than 6 hours extubations after surgery.

The effects of different extubation times on the UTI and urinary retention on patients with ureteral intubation were analyzed. The publication bias of the 4 trials included using Nfs suggested that there is less likelihood of publication bias.

In a previous study,^[7]^ it was demonstrated that the removal of urethral catheter immediately after gynecologic surgery could reduce the incidence of UTI, but with higher incidence of recatheterization. A possible reason was anesthesia which led to dysuria in patients undergoing gynecologic surgery, and thus, immediate catheter removal easily caused urinary retention. A prospective study conducted by Lamonerie et al^[21]^ confirmed that spinal anesthesia is a risk factor for urinary retention after surgery.

Our review found that compared with immediately catheter removal, extubation within 6 hours after surgery decreased the incidence of urinary retention, although there was no significant difference with respect to the incidence of UTI. The most possible explanation is that it takes an average of 4 hours to 5 hours after spinal anesthesia to recover bladder function.^[1]^ Therefore, it is reasonable to suggest that the timing of removal of urinary catheter should be within 6 hours after surgery, when compared with immediately catheter removal. While in the comparison between ≤6  hours extubation group and >6 hours extubation group, we included 2 papers^[1,18]^ that included the transvaginal repairing surgery of vaginal prolapse which may hinders normal urinary voiding, so we conduct the subgroup analysis to explore the clinical differences among the included studies. It showed that the incidences of urinary retention were similar in the gynecologic surgery excluded the vaginal surgery group and vaginal surgery group.

Indwelling urinary catheter is commonly used after gynecological surgery to assess urine output and to prevent postoperative urinary retention.^[22]^ When a urinary catheter is inserted into the urethra and placed in the bladder, it can damage the urethra and bladder mucosa. It can also carry urethral organisms which may be bacteria into the bladder,^[23]^ reduce the ability of the defense against harmful bacteria, thereby increasing the incidence of UTI. In this review, we found that urinary catheter removal >6 hours after surgery was associated with a significant higher incidence of UTI. The reasons might be that indwelling urinary catheter damages the urethra and bladder mucosa in the intubation process, and with the prolonged time of the indwelling catheter, it can attract colonization of biofilm microorganisms that have the ability to withstand drying, ultraviolet radiation, and antimicrobial agents.^[24]^ In addition, with prolonged postoperative catheterization, the urinary catheter organism forms biofilms on the catheter surface that contribute to the infection process and make this biofilms difficult to eradicate using antibiotics and cause the occurrence of UTI.^[25]^ It has been established that the duration of urinary catheterization is one of the important risk factor for the development of UTI.^[7]^ Therefore, to some extent, removal time of the urinary catheter at ≤6 hours seems to be suitable for patients, when compared with >6 hours catheter removal.

There are some limitations in the current study. The review only included 8 trials which limited the production of funnel plots to show publication bias. Three studies^[12,14,17]^ showed the drawback of methodological quality, especially in allocation concealment. The heterogeneity of included studies was existed regarding the difference in the type of surgeries, degree of invasiveness and type of anesthesia, which may result in different extubation time. In 8 articles included in this study, 5 articles have reported that patients who had UTI preoperatively confirmed by urine analysis culture were excluded, the remaining 3 articles did not mention the problem of preoperative urine culture. Due to the limited number of articles, the remaining 3 articles were included in this study, which may limit the interpretation of results. More trials are needed to confirm the results of this review and more studies of catheter removal within 6 hours should be explored to find out the most reasonable time in order to reduce the risk of UTI.

In summary, the results of this meta-analysis showed that extubation within 6 hours after gynecologic surgery was associated with lower risks of urinary retention and UTI, when compared with immediately and >6 hours catheter removal, respectively. It is recommended that urinary catheter extubation within 6 hours postoperatively was the appropriate timing for patients undergoing gynecologic surgery which excluded the vaginal surgery.

## Author contributions

**Conceptualization:** Lan Gu.

**Data curation:** Hui Huang, Li Dong.

**Formal analysis:** Hui Huang, Li Dong.

**Funding acquisition:** Lan Gu.

**Investigation:** Hui Huang, Li Dong.

**Methodology:** Hui Huang, Li Dong.

**Project administration:** Lan Gu.

**Resources:** Hui Huang, Li Dong.

**Software:** Hui Huang, Li Dong.

**Supervision:** Lan Gu.

**Writing – original draft:** Li Dong, Lan Gu.

**Writing – review and editing:** Lan Gu.
